# Does a Train-the-Trainer Approach for Enhancing Healthcare Professionals' Skills in Patient Education during Inpatient Medical Rehabilitation Improve Patient Outcomes?

**DOI:** 10.1155/2020/8316256

**Published:** 2020-03-25

**Authors:** Anneke Ullrich, Laura Inhestern, Jochen Wehrmann, Martin Raida, Matthias Köhler, Corinna Bergelt

**Affiliations:** ^1^Department of Medical Psychology, Center for Psychosocial Medicine, University Medical Center Hamburg-Eppendorf, Martinistr. 52, 20246 Hamburg, Germany; ^2^VAMED Rehabilitation Clinic Bad Berleburg, Arnikaweg 1, 57319 Bad Berleburg, Germany; ^3^VAMED Rehabilitation Clinic Bergisch-Land, Im Saalscheid 5, 42369 Wuppertal, Germany; ^4^VAMED Rehabilitation Clinic Damp, Seute Deern Ring 30, 24351 Ostseebad Damp, Germany

## Abstract

This study is aimed at identifying the impact of a team-based train-the-trainer program (TTT-P) to enhance healthcare professional (HCP) skills in patient education during medical rehabilitation. Focusing on patient-reported outcomes, a prospective, sequential two-cohort study was conducted in the fields of psychosomatic and oncological rehabilitation. Two hundred fifteen patients were evaluated before (Cohort 1) and 196 post implementation of TTT-P (Cohort 2). Patients of both cohorts completed validated questionnaires on self-management (heiQ®), general self-efficacy (GSE scale), and quality of life (WHOQOL-Bref) at the beginning, at the end, and at the 6-month follow-up to analyze short- and intermediate-term effects. Analyses were conducted separately for the psychosomatic and oncological setting. Results showed that TTT-P had no impact on patient outcomes in both rehabilitation settings. Patients did report positive outcomes as a result of the whole inpatient rehabilitation programs, though effects at follow-up were mostly small to medium size. Concerning self-management competencies, cancer patients gained less benefit during rehabilitation than psychosomatic patients. In conclusion, TTT-P did not result in measurable improvements at the patient level, likely because of the limited nature of the intervention. However, these populations of rehabilitants took benefit from participating in a multimodal rehabilitation program, of which patient education is one part.

## 1. Introduction

Chronic illnesses have great impact on the patients' lives, regarding both physical and mental health. Patient self-management has been proposed to be an effective strategy to manage the chronic effects of illness, i.e., in cancer [1]. Self-management of the disease, empowerment in making informed choices, development of coping strategies, and modification of health behavior are important goals of patient education interventions [2, 3]. Meta-analyses found patient education to be an effective and cost-saving approach [4–6]. In the context of medical rehabilitation, effectiveness of standardized patient education programs has been evaluated for various medical conditions, i.e., heart failure, asthma, chronical back pain, or fibromyalgia [7–9].

Delivering quality patient education demands specific knowledge on medical conditions and their treatment, models of health psychology, and factors determining health-related behavior [10]. Patient educators need specific didactical qualification and have to coordinate and organize patient education programs within their health organization [11]. Further, skills in creating and maintaining a positive learning atmosphere and dealing with difficult (group) situations are necessary [12]. Competencies of healthcare professionals (HCP) educating patients are assumed to influence the quality and effectiveness of patient education measures [13]. Yet, few healthcare disciplines received formal training for patient education during vocational education [14], and the quality of patient education interventions may be impeded by educators' lacking knowledge on patient-oriented didactics [15]. For filling this gap, capacity building approaches for patient educators include train-the-trainer programs [15, 16], which are based on the idea that experts train those who will deliver the intervention to enhance the trainees' knowledge and skills.

Patient education provided by HCP of different disciplines plays an important role within German medical rehabilitation [17, 18]. According to German Social Law (SGB IX), patients are legally entitled to access rehabilitation programs when meeting specific criteria [19]. Such multimodal programs are mostly provided by certified inpatient rehabilitation clinics and are based on a biopsychosocial approach as outlined in the International Classification of Functioning, Disability and Health (ICF) [20]. A survey on patient education practice among 900 German rehabilitation clinics indicated a lack of quality requirements, including trainings for HCP who provide patient education [21]. Qualified training for HCP educating patients has been identified to be of high importance by stakeholders and HCP alike [13, 22].

Though train-the-trainer approaches are widely used and may be considered to be an effective strategy to enhance HCP skills in patient education, the impact of such an approach on patient outcomes has rarely been examined. However, a narrative review indicated that improvements of patient outcomes could be gained by implementation of train-the-trainer programs [16]. Our study is aimed at evaluating the effects of a train-the-trainer approach for HCP educating patients in the setting of German oncological and psychosomatic inpatient rehabilitation with focus on improvements of patient outcomes. We hypothesized that outcomes of patients being educated after implementation of a train-the-trainer program (TTT-P) are better than after usual care regarding self-management competence, which was defined as the primary outcome. Furthermore, we hypothesized better performance in general self-efficacy and quality of life (QoL) as secondary outcomes.

## 2. Materials and Methods

### 2.1. Study Design and Procedure

We conducted a multicentered, prospective, sequential pre-post cohort study in two rehabilitation clinics in Germany, one specialized in oncological rehabilitation and one in psychosomatic inpatient rehabilitation. Patients treated before implementation of TTT-P (Cohort 1) were compared to those treated post implementation (Cohort 2). Cohort 1 in the psychosomatic sample included patients admitted from 26 April 2014 to 5 October 2014 and Cohort 2 those admitted from 27 January 2015 to 30 December 2015. Cohort 1 in the oncological sample included patients admitted from 23 September 2014 to 5 December 2014, and Cohort 2 those admitted from 26 October 2015 to 24 November 2016. In both settings, a waiting period elapsed after HCP had received TTT-P to avoid overlap of patients of Cohort 1 at the onset of Cohort 2. Patients were unaware to which group they belonged.

Patients were consecutively recruited by the treating rehabilitation physician or psychologist during the first clinical encounter. Inclusion criteria were an ICD-10 diagnosis of cancer or psychosomatic/psychiatric disorder (depending on the rehabilitation setting) and being aged ≥18 years. Exclusion criteria were cognitive impairment, insufficient German, or severe psychological/physical stress (assessed by the treating physician/psychologist).

Data were collected at the beginning of the rehabilitation program, at the end, and at the 6 months post. During rehabilitation, patient questionnaires were handed out at the first and last clinical encounters with physicians/psychologists. The follow-up questionnaire was mailed together with a return envelope including a single reminder after four weeks. Medical data were collected from the treating physician/psychologist by means of a short standardized form and from routine data. In each clinic, a person was determined who was responsible for equipping physicians/psychologists with study material, monitoring data collection during rehabilitation, and managing data collection at the follow-up.

The study was approved by the ethics committees of the General Medical Councils of Hamburg, Northrhine and Westphalia-Lippe. Written informed consent was obtained from all study participants.

### 2.2. Intervention

#### 2.2.1. Train-the-Trainer Program

The TTT-P received by HCP is aimed at enhancing skills requested for quality patient education. At an interval of approximately three months, it comprised two 2-day seminars (in-house) and was provided by an external organization specialized in TTT-P for patient educators in the health sector. Thus, it was carried out independently from the research group evaluating the intervention. It followed an interprofessional, team-based approach and was designed for a general health education program for patients receiving inpatient medical rehabilitation, irrespective of indications. Content of the TTT-P was modules addressing (a) leading and moderating groups, (b) activating and motivating patients, and (c) use of didactical methods.

Overall, 29 HCP participated in the TTT-P, of which 11 worked in the psychosomatic rehabilitation clinic and 18 in the oncological rehabilitation clinic. Regarding disciplines, most HCP were psychosocial staff (44%), followed by physicians (35%), nursing staff and physical therapists (9%), and others (13%). In 63% of HCP, the work experience in the current occupational field was >5 years. Before implementation of the TTT-P, 13% reported little experience in patient education, whereas 29% had 1 to 5 years of experience, 21% > 5 to 10 years, and 38% > 10 years. The weekly workload covering patient education measures during working hours was mean 17.1% (SD 23.4; range: 2-80%). The same HCP continued to educate patients after implementation of the TTT-P.

#### 2.2.2. Rehabilitation Programs for Patients (Non Study-Specific)

Patients received a rehabilitation program with high treatment intensity. Clinics are staffed with multidisciplinary HCP including physicians, nursing staff, physical therapists/sport teachers, psychologists/other psychosocial therapists, social workers, and nutritionists. Oncological rehabilitation on average lasts 3 weeks and is mostly initiated after completion of primary treatment. It comprises medical treatment, physical training, psychological support/therapy, and social counseling [23]. Psychosomatic rehabilitation on average lasts 5 weeks involving multilevel psychosomatic assessment and multimodal treatment. A majority of patients is sent to inpatient treatment after continuing sick leave or application for early retirement [24].

### 2.3. Outcomes and Measurements

The primary outcome was self-management competencies at the end of the rehabilitation program (short-term effects). Secondary outcomes were general self-efficacy and QoL (short-term effects) and self-management competencies, self-efficacy, and QoL at the 6-month follow-up (intermediate-term effects). We used validated and reliable self-report questionnaires for measurement of outcomes.

The German version of the Health Education Impact Questionnaire (heiQ®) was applied to measure self-management [25, 26]. The heiQ® is a generic instrument comprising 40 items, each rated on a 4-point Likert scale. The eight independent dimensions of the heiQ® are “positive and active engagement in life,” “health-directed behavior,” “skill and technique acquisition,” “constructive attitudes and approaches,” “self-monitoring and insight,” “health service navigation,” “social integration and support,” and “emotional well-being.” Except for “emotional well-being,” higher values indicate better self-management competencies.

For measurement of self-efficacy, the general self-efficacy (GSE) scale [27] was applied. The instrument comprises 10 items assessed on a 4-point Likert scale. A summary score (possible range: 4-40) informs about the extent of general self-efficacy, with higher values indicating higher self-efficacy.

QoL was assessed using the WHOQOL-Bref [28], which is a 26-item version of the WHOQOL-100 assessment. Items are rated on 5-point Likert scales. The instrument comprises six domains: “physical health,” “psychological health,” “social relations,” “environment,” “global quality of life” (single item), and “satisfaction with quality of life” (single item). Scores are transformed on a scale from 0 to 100. For single-item scales, scores range from 1 to 5. Higher scores represent higher levels of quality of life.

To gain insights into beneficial aspects of patient education, patients answered an open-ended question at the end of the rehabilitation program: “Which aspects of patient education did you perceive as most helpful? Please describe.” Participants could note as many aspects as wanted.

Information on sociodemographic variables was collected from patients, and medical data were obtained from the treating physicians/psychologists. The dose of patient education during rehabilitation (therapeutic treatment category “information, motivation, and education,” as constituted in the Pension Insurance's KTL classification system [29]) was derived from routine data.

### 2.4. Data Analysis

Due to diverging sample characteristics, effects of the TTT-P were analyzed separately for the psychosomatic and oncological rehabilitation settings. Short-term effects (end of the rehabilitation program) and intermediate-term effects (6-month follow-up) were evaluated using analysis of variance (ANOVA) with repeated measures. Effect sizes for time, group (Cohort 1, Cohort 2), and interaction effects (time∗group) were calculated by partial eta^2^ (small effect = 0.01, medium effect = 0.06, and large effect = 0.14).

Transcripts of aspects that were perceived as helpful in the context of patient education were first coded and analyzed using qualitative content analysis to identify key themes. In total, 141 psychosomatic patients and 97 cancer patients responded to the question. All transcripts of beneficial aspects were coded for the presence or absence of each of the five themes identified. Group differences between frequencies of emerging themes (Cohort 1, Cohort 2) were analyzed using chi-square tests. All analyses were performed using the statistical package SPSS version 18.0 (SPSS Inc., Chicago, Illinois).

## 3. Results

### 3.1. Sample Development, Sample Characteristics, and Nonresponder Analysis

#### 3.1.1. Sample Development

**Figure 1:**
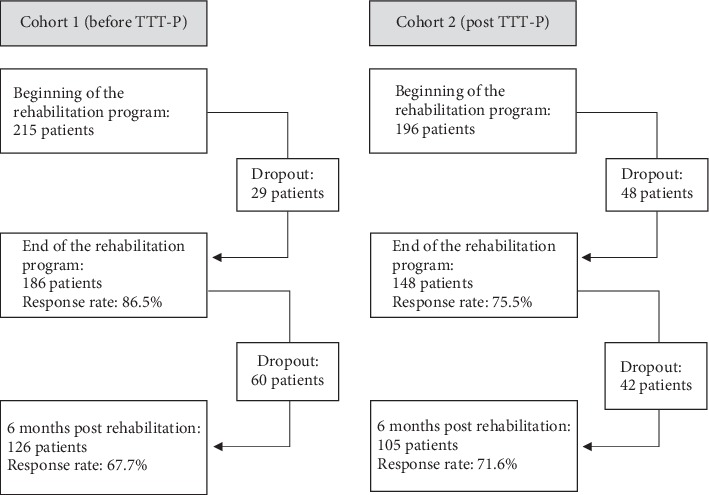
Sample development of the two cohorts before and after implementation of the train-the-trainer program (TTT-P) for healthcare professionals.

Overall, 411 patients (215 in Cohort 1 and 196 in Cohort 2, post TTT-P) completed the first, 334 (81.3%) second, and 232 (69.5%) third assessment (Figure 1). Data collection started in April 2014, and the follow-up for Cohort 2 was completed in August 2017.

#### 3.1.2. Sample Characteristics

Of 220 psychosomatic patients, 49% belonged to Cohort 1 and 51% to Cohort 2. Across cohorts, mean age was in the midforties, one-third were male, and approximately half of the patients were diagnosed with affective disorders. Of 191 cancer patients, 57% belonged to Cohort 1 and 42% to Cohort 2. In both, half of the patients were male, mean age was about 60 years, and approximately 40% had cancer of the digestive organs. Details on patient characteristics are reported in Table 1.

#### 3.1.3. Nonresponder Analyses at the 6-Month Follow-Up

On average, respondents in both settings were older than nonrespondents at the first assessment (psychosomatic: 47.6 vs. 41.3 years, *p* = .001; oncological: 62.6 vs. 58.6 years, *p* = .017). Further, respondents of the oncological sample had reported less depressive symptoms on the Hospital Anxiety and Depression Scale (HADS) than nonrespondents (depression subscale ≤ 7 points: 77% vs. 49%, *p* = .001).

### 3.2. Extent of Patient Education Received during Medical Rehabilitation

We performed subgroup analyses of 157 psychosomatic and 152 cancer patients, for whom detailed information on therapeutic treatment was available.

Among the psychosomatic sample, the delivered dose of procedures from the KTL category “information, motivation, and education” was average 1.4 ± 0.7 hours per week with 100% of patients receiving such interventions. Cohorts were comparable with regard to the received dose of interventions (Cohort 1: *n* = 90, mean 1.4 ± 0.6; Cohort 2, post TTT-P: *n* = 67, mean 1.4 ± 0.6; *p* = .964).

Among the oncological sample, the delivered dose of therapeutic procedures from the respective KTL category was average 3.9 ± 1.9 hours per week with 100% of patients receiving such interventions. Although a slightly higher dose of interventions was documented for patients post TTT-P, no significant group difference was observed (Cohort 1: *n* = 95, mean 3.7 ± 1.7; Cohort 2, post TTT-P: *n* = 57, mean 4.2 ± 2.2; *p* = .126).

### 3.3. Primary Outcome: Short-Term Effects on Self-Management Competencies

Descriptive data and ANOVA results on short-term-effects of the TTT-P are presented in Table 2 for psychosomatic patients and in Table 3 for cancer patients. In both rehabilitation settings, analyses revealed no significant group differences (Cohort 1, Cohort 2) with regard to changes in short-term self-management.

Patients of each setting reported improved self-management at the end of the rehabilitation program. Most self-management competency scores increased significantly among psychosomatic patients, except for “health service navigation” (*p* < .01, medium to large effect sizes). Cancer patients improved slightly but significantly in “positive and active engagement in life,” “health-directed activities,” and “skill and technique acquisition” (*p* < .01, small effect sizes).

### 3.4. Secondary Outcomes: Short-Term Effects on Self-Efficacy and Quality of Life

Detailed data is displayed in Table 2 for psychosomatic patients and in Table 3 for cancer patients. In both settings, no significant interaction effects between time and group (Cohort 1, Cohort 2) emerged, neither for general self-efficacy nor for QoL.

During the rehabilitation program, general self-efficacy significantly improved in psychosomatic patients (*p* ≤ .001, large effect size) but declined in cancer patients (*p* = .014, small effect size). Psychosomatic patients reported improved QoL outcomes across all scales (*p* ≤ .001, medium to large effect sizes) and cancer patients for all scales except for “environment” (*p* ≤ .001, small effect sizes).

### 3.5. Secondary Outcomes: Intermediate-Term Effects on Self-Management, Self-Efficacy, and Quality of Life

Descriptive data and ANOVA results on intermediate-term effects of the TTT-P are presented in Table 4 for psychosomatic patients and in Table 5 for cancer patients. In both settings, we found no significant interaction effects between time and group (Cohort 1, Cohort 2) in any outcome scale at the 6-month follow-up. Aside from the lacking impact of TTT-P, improved patient outcomes tended to persist at the follow-up, although effects were small sized.

Regarding self-management competencies, changes over time were significant across all scales but “health service navigation” in psychosomatic patients (*p* < .05, small to large effect sizes). In cancer patients, changes were only significant in two out of eight scales: “positive and active engagement in life” and “skill and technique acquisition” (*p* < .05, small effect sizes). We found significant time effects for general self-efficacy in psychosomatic patients (*p* ≤ .001, medium effect size), but not in the oncological sample. Changes of QoL scores revealed to be significant for all scales in psychosomatic patients (*p* < .01, medium to large effect sizes). With exception of “environment,” this also applied for cancer patients (*p* < .01, small to medium effect sizes).

### 3.6. Aspects Regarded Helpful during Patient Education

**Figure 2:**
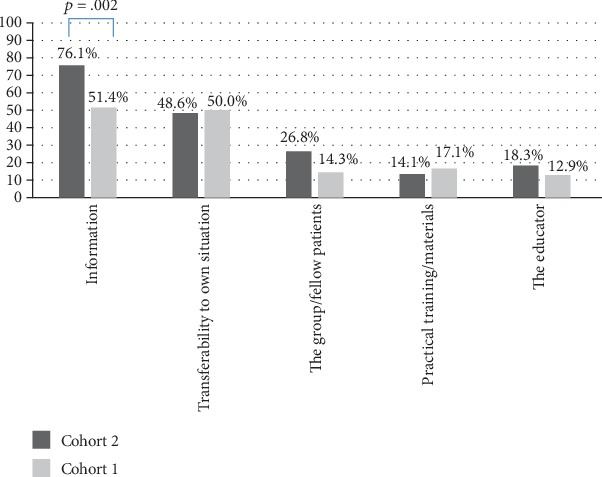
Aspects regarded most helpful in the context of patient education during psychosomatic rehabilitation (*N* = 141; Cohort 2: post train-the-trainer program).

**Figure 3:**
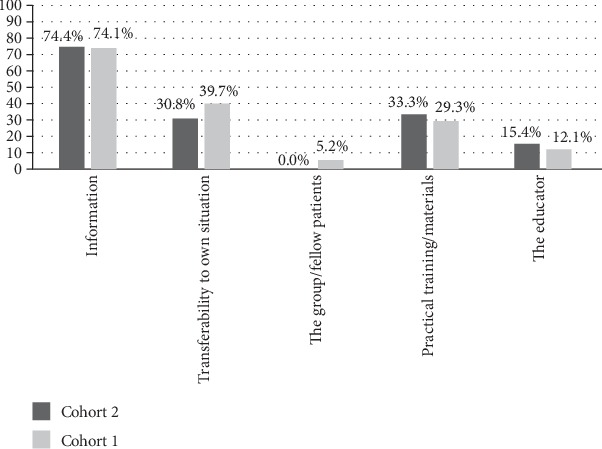
Aspects regarded most helpful in the context of patient education during oncological rehabilitation (*N* = 97; Cohort 2: post train-the-trainer program).

Within qualitative content analysis, five key themes emerged: “information,” “transferability to own situation,” “the group/fellow patients,” “practical training/materials,” and “the educator” were identified to be the most helpful aspects of patient education. Among 141 psychosomatic patients, who at least indicated one aspect, “information” was significantly more often reported by patients of Cohort 2 (post TTT-P) compared with Cohort 1 (Figure 2). Patient cohorts of the oncological sample did not differ significantly (Figure 3). Irrespective of rehabilitation setting and group (Cohort 1, Cohort 2), “information” was the most prevalent aspect, followed by “transferability to own situation.”

## 4. Discussion

Patient education plays an important role in medical rehabilitation programs. Its goal is to help patients improve their knowledge, skills, and motivation and to engage them in coping with their disease [3]. HCP who provide patient education need a broad range of qualifications [11, 12], and a TTT-P was implemented to support HCP in developing and strengthening their competencies.

Our study showed that rehabilitation outcomes did not differ before (Cohort 1) and post (Cohort 2) implementation of the TTT-P, neither at the end of the rehabilitation program nor at the 6-month follow-up. Thus, our hypotheses that patients receiving rehabilitation after the HCPs' training would report better self-management competencies, self-efficacy, and QoL could not be validated. The only difference between cohorts was found in the qualitative analysis of aspects of patient education that were perceived as helpful. Here, “information” was more often mentioned by patients of Cohort 2, but this finding only applied to psychosomatic patients.

One possible explanation is that patient education is a small part of the 3-5-week lasting, multicomplex rehabilitation programs that patients received. The main goals of medical rehabilitation programs are improvement of the patient's medical condition, development of coping strategies and reintegration into social and working life with the ICF [20] serving as a conceptual basis. The resulting complexity of rehabilitation programs equally applies to the psychosomatic and cancer rehabilitation settings [23, 24]. Although it has been indicated that staff education may impact patient outcomes (train-the-trainer in acute clinical settings) [16], we were not able to detect effects in multifactorial inpatient rehabilitation. Studies examining the direct effect of patient education interventions in medical rehabilitation programs found no systematic effects of the implemented patient education curricula when comparing the intervention and control groups [7–9]. Further, in our study, all patients received patient education with “information, motivation, and education” constituting a particular category of therapeutic treatment in the Pension Insurance's KTL classification system [29]. This underlines the high relevance given to patient education already prior to the implementation of the TTT-P. However, it also raises the question if additional effects of a train-the-trainer approach are noticeable for patients who regularly perceive patient education as part of a rehabilitation program.

Apart from these findings, our results show that patient outcomes improved short term (from the beginning to the end of the rehabilitation program), which might indicate the effectiveness of the rehabilitation programs. Patients of both settings reported better self-management competencies, but in psychosomatic patients, stronger and more systematic effects of time were found. Short-term QoL also significantly improved in both settings, with exception of “environment” in cancer patients. Fostering self-management competencies and enabling patients' reintegration into social life, despite disabilities and side effects limiting the patients' QoL, are an important goal of rehabilitation programs [19]. Therefore, effects obtained are highly relevant. At the 6-month follow-up, outcomes tended to persist when compared to the end of the rehabilitation program. Considering all time points included in the study, we observed more and stronger time effects in psychosomatic patients. Since found effects of the whole inpatient rehabilitation programs were mostly small to medium size, it is unlikely to detect significant effects due to changes in the patient education approach, with patient education sessions being one element of rehabilitation.

Some limitations of the study should be noted. First, systematic collection of data on nonparticipants was not feasible due to organizational reasons, and a selection bias towards patients with better physical and/or mental health and those with higher motivation is possible. Thus, data has to be interpreted carefully with respect to generalization, while satisfying response rates within the study sample strengthen robustness of data. Second, we conducted a sequential pre-post cohort study design with each rehabilitation clinic serving as its own control. While having advantages (i.e., reducing heterogeneity), the design might have caused pitfalls such as selection bias, and a parallel-group study design using a matched comparison group of patients from nonintervention rehabilitation clinics might be considered for future research.

Third, the intervention was embedded with patient education, which is part of all rehabilitation programs in Germany, and differences of the patient education procedures before and after the TTT-P were not measured. Fourth, knowledge is missing if to which effect HCP could use trained skills, i.e., because of large patient groups, time limitations, or other impeding factors, or if they used them already before. Further, we do not know how many patients were educated by different disciplines and/or HCP; thus, possible imbalances and its effects cannot be assessed. These missing but viable data limit the ability to answer our research question, and future research should address these methodological shortcomings.

## 5. Conclusion

In conclusion, our results underscore the possible effects of comprehensive, multidisciplinary medical rehabilitation programs on patients' self-management competencies, self-efficacy, and QoL, particularly in the short term. We addressed the question if effectiveness of a train-the-trainer approach could be measured at the level of patient outcomes, basing on the requirement that measuring effectiveness of any clinical activity needs to incorporate measures of the direct impact on the patient's health [30]. However, effects of a TTT-P to enhance HCPs' educating skills on patient outcomes could not be confirmed. Further studies need to investigate the impact of a train-the-trainer approach on educator-reported outcomes.

## Figures and Tables

**Table 1:** Baseline characteristics of the samples.

	Cancer patients		Psychosomatic patients	
	Cohort 1 (*N* = 108)	Cohort 2 (*N* = 83)	Cohort 1 (*N* = 107)	Cohort 2 (*N* = 113)
	*n* (%)	*n* (%)	*n* (%)	*n* (%)
Male	47 (51.1)	30 (50.0)	31 (34.1)	25 (28.4)
Age in years, mean (SD)	62.2 (10.7)	60.1 (9.9)	44.3 (11.0)	47.0 (10.3)
Marital status				
Single	12 (13.0)	9 (15.0)	29 (32.2)	23 (26.1)
Married	54 (58.7)	46 (76.7)	36 (40.0)	45 (51.1)
Other	26 (28.3)	5 (8.3)	25 (27.8)	20 (22.7)
Education				
≤9 years	47 (52.2)	30 (52.6)	22 (24.2)	13 (14.8)
10-12 years	21 (23.3)	11 (19.3)	33 (36.3)	40 (45.5)
13 years	22 (24.4)	16 (28.1)	36 (39.6)	35 (39.8)
Occupational position				
Manual worker	26 (27.4)	18 (30.0)	12 (13.2)	14 (15.9)
White-collar job	52 (54.7)	29 (48.3)	65 (71.4)	63 (71.6)
Self-employed or public servant	12 (11.3)	11 (18.3)	5 (5.5)	6 (6.8)
Cancer diagnosis				
Digestive organs	33 (38.8)	18 (36.7)	n.a.	n.a.
Respiratory organs	15 (17.6)	6 (12.2)	n.a.	n.a.
Breast	9 (10.6)	7 (14.3)	n.a.	n.a.
Other	18 (33.0)	28 (36.8)	n.a.	n.a.
Time since cancer diagnosis				
<3 months	38 (40.9)	30 (51.7)	n.a.	n.a.
4-6 months	13 (14.0)	7 (12.1)	n.a.	n.a.
≥7 months	42 (45.2)	21 (36.2)	n.a.	n.a.
Psychiatric disorder				
Affective	n.a.	n.a.	43 (47.8)	40 (46.0)
Neurotic/stress-related/somatoform	n.a.	n.a.	13 (14.4)	22 (25.3)
Personality/behavioral	n.a.	n.a.	14 (5.6)	8 (9.2)
Other	n.a.	n.a.	17 (19.4)	20 (22.2)
Duration of symptoms				
<1 year	n.a.	n.a.	12 (14.5)	13 (14.8)
1-5 years	n.a.	n.a.	35 (42.2)	32 (36.3)
>5 years	n.a.	n.a.	63 (43.3)	56 (48.9)

Abbreviations: SD: standard deviation; n.a.: not applicable.

**Table 2:** Short-term effects on primary and secondary outcomes in psychosomatic patients (*n* = 179).

Psychosomatic patients	Beginning of the rehabilitation program	End of the rehabilitation program	Time		Time∗group	
	Mean (SD)	Mean (SD)	*p* value	Partial eta^2^	*p* value	Partial eta^2^
Self-management competence (heiQ®)						
Positive and active engagement in life						
Cohort 1	2.6 (0.6)	2.9 (0.7)	**<.001**	.175	.898	.000
Cohort 2 (post TTT-P)	2.6 (0.7)	2.8 (0.7)				
Health-directed activities						
Cohort 1	2.5 (0.9)	2.8 (0.8)	**<.001**	.234	.230	.008
Cohort 2 (post TTT-P)	2.5 (0.9)	2.9 (0.8)				
Skill and technique acquisition						
Cohort 1	2.7 (0.6)	2.9 (0.5)	**<.001**	.138	.379	.004
Cohort 2 (post TTT-P)	2.6 (0.5)	2.9 (0.6)				
Constructive attitudes and approaches						
Cohort 1	2.9 (0.7)	3.0 (0.6)	**<.001**	.090	.733	.001
Cohort 2 (post TTT-P)	2.8 (0.7)	3.0 (0.8)				
Self-monitoring and insight						
Cohort 1	2.9 (0.5)	3.0 (0.5)	**<.001**	.160	.178	.010
Cohort 2 (post TTT-P)	2.8 (0.4)	3.1 (0.4)				
Health service navigation						
Cohort 1	3.2 (0.5)	3.2 (0.6)	.259	.007	.252	.008
Cohort 2 (post TTT-P)	3.1 (0.4)	3.2 (0.5)				
Social integration and support						
Cohort 1	2.6 (0.7)	2.7 (0.7)	**.002**	.056	.468	.003
Cohort 2 (post TTT-P)	2.6 (0.6)	2.8 (0.7)				
Emotional distress						
Cohort 1	2.6 (0.8)	2.3 (0.8)	**<.001**	.171	.721	.001
Cohort 2 (post TTT-P)	2.6 (0.7)	2.3 (0.8)				
General self-efficacy (GSE)						
Cohort 1	23.5 (6.4)	25.9 (6.8)	**<.001**	.169	.591	.002
Cohort 2 (post TTT-P)	23.7 (6.1)	25.8 (5.9)				
Health-related quality of life (WHOQOL-Bref)						
Physical health						
Cohort 1	50.4 (19.0)	62.9 (21.5)	**<.001**	.397	.203	.010
Cohort 2 (post TTT-P)	53.5 (19.4)	63.3 (21.8)				
Psychological health						
Cohort 1	41.3 (21.0)	56.2 (21.6)	**<.001**	.437	.215	.009
Cohort 2 (post TTT-P)	46.9 (21.7)	58.9 (24.2)				
Social relations						
Cohort 1	53.1 (22.8)	61.9 (21.3)	**<.001**	.130	.256	.008
Cohort 2 (post TTT-P)	54.3 (24.0)	59.8 (22.9)				
Environment						
Cohort 1	64.2 (14.3)	68.8 (15.8)	**<.001**	.102	.250	.008
Cohort 2 (post TTT-P)	67.2 (15.1)	69.8 (14.5)				
Global quality of life						
Cohort 1	2.9 (1.0)	3.4 (1.0)	**<.001**	.281	.851	.000
Cohort 2 (post TTT-P)	2.9 (0.9)	3.5 (.9)				
Satisfaction with quality of life						
Cohort 1	2.4 (1.1)	3.2 (1.1)	**<.001**	.420	.848	.000
Cohort 2 (post TTT-P)	2.3 (1.0)	3.2 (1.1)				

Abbreviations: SD: standard deviation; TTT-P: train-the-trainer program for healthcare professionals. Significant *p* values are marked in bold.

**Table 3:** Short-term effects on primary and secondary outcomes in cancer patients (*n* = 155).

Cancer patients	Beginning of the rehabilitation program	End of the rehabilitation program	Time		Time∗group	
	Mean (SD)	Mean (SD)	*p* value	Partial eta^2^	*p* value	Partial eta^2^
Self-management competence (heiQ®)						
Positive and active engagement in life						
Cohort 1	3.1 (0.5)	3.2 (0.6)	**.013**	.042	.807	.000
Cohort 2 (post TTT-P)	3.0 (0.6)	3.1 (0.5)				
Health-directed activities						
Cohort 1	3.0 (0.8)	3.1 (0.7)	**.013**	.042	.337	.006
Cohort 2 (post TTT-P)	3.0 (0.8)	3.1 (0.6)				
Skill and technique acquisition						
Cohort 1	3.0 (0.5)	3.0 (0.5)	**.041**	.028	.459	.004
Cohort 2 (post TTT-P)	3.0 (0.6)	3.1 (0.5)				
Constructive attitudes and approaches						
Cohort 1	3.4 (0.6)	3.3 (0.5)	.126	.016	.834	.000
Cohort 2 (post TTT-P)	3.4 (0.6)	3.4 (0.5)				
Self-monitoring and insight						
Cohort 1	3.1 (0.5)	3.2 (0.4)	.055	.025	.188	.012
Cohort 2 (post TTT-P)	3.1 (0.4)	3.1 (0.4)				
Health service navigation						
Cohort 1	3.4 (0.5)	3.3 (0.5)	.923	.000	.170	.013
Cohort 2 (post TTT-P)	3.4 (0.5)	3.4 (0.4)				
Social integration and support						
Cohort 1	3.2 (0.6)	3.2 (0.6)	.698	.001	.362	.006
Cohort 2 (post TTT-P)	3.3 (0.7)	3.2 (0.6)				
Emotional distress						
Cohort 1	2.3 (0.7)	2.2 (0.7)	.285	.008	.129	.016
Cohort 2 (post TTT-P)	2.3 (0.8)	2.3 (0.7)				
General self-efficacy (GSE)						
Cohort 1	29.4 (4.6)	29.1 (5.4)	**.014**	.040	.070	.022
Cohort 2 (post TTT-P)	29.1 (5.3)	27.6 (6.3)				
Health-related quality of life (WHOQOL-Bref)						
Physical health						
Cohort 1	61.9 (18.8)	67.5 (17.9)	**<.001**	.216	.828	.000
Cohort 2 (post TTT-P)	61.7 (18.6)	66.9 (17.1)				
Psychological health						
Cohort 1	68.2 (16.0)	71.6 (16.6)	**<.001**	.091	.800	.000
Cohort 2 (post TTT-P)	65.3 (20.2)	68.3 (19.0)				
Social relations						
Cohort 1	69.9 (18.5)	73.2 (18.2)	**<.001**	.092	.456	.004
Cohort 2 (post TTT-P)	67.9 (21.7)	72.8 (20.3)				
Environment						
Cohort 1	71.7 (14.7)	72.1 (13.7)	.078	.022	.279	.008
Cohort 2 (post TTT-P)	72.5 (14.6)	74.4 (13.1)				
Global quality of life						
Cohort 1	3.3 (.8)	3.6 (0.8)	**<.001**	.167	.346	.006
Cohort 2 (post TTT-P)	3.3 (.8)	3.6 (0.7)				
Satisfaction with quality of life						
Cohort 1	3.0 (1.0)	3.3 (0.9)	**<.001**	.175	.295	.007
Cohort 2 (post TTT-P)	2.9 (1.0)	3.4 (0.8)				

Abbreviations: SD: standard deviation; TTT-P: train-the-trainer program for healthcare professionals. Significant *p* values are marked in bold.

**Table 4:** Intermediate-term effects on secondary outcomes in psychosomatic patients (*n* = 122).

Psychosomatic patients	Beginning of the rehabilitation program	End of the rehabilitation program	6-month follow-up	Time		Time∗group	
	Mean (SD)	Mean (SD)	Mean (SD)	*p* value	Partial eta^2^	*p* value	Partial eta^2^
Self-management competence (heiQ®)							
Positive and active engagement in life							
Cohort 1	2.5 (0.5)	2.8 (0.7)	2.8 (0.7)	**<.001**	.113	.208	.013
Cohort 2 (post TTT-P)	2.6 (0.7)	2.9 (0.7)	2.7 (0.7)				
Health-directed activities							
Cohort 1	2.6 (0.8)	2.9 (0.7)	2.8 (0.7)	**<.001**	.170	.209	.013
Cohort 2 (post TTT-P)	2.4 (0.9)	2.9 (0.9)	2.8 (0.8)				
Skill and technique acquisition							
Cohort 1	2.6 (0.6)	2.8 (0.5)	2.9 (0.6)	**<.001**	.148	.449	.007
Cohort 2 (post TTT-P)	2.6 (0.6)	2.9 (0.6)	2.9 (0.5)				
Constructive attitudes and approaches							
Cohort 1	2.8 (0.7)	3.0 (0.6)	3.0 (0.7)	**<.001**	.071	.151	.016
Cohort 2 (post TTT-P)	2.9 (0.7)	3.0 (0.7)	2.9 (0.7)				
Self-monitoring and insight							
Cohort 1	2.8 (0.5)	3.0 (0.5)	3.0 (0.4)	**<.001**	.130	.323	.010
Cohort 2 (post TTT-P)	2.9 (0.4)	3.0 (0.4)	3.1 (0.5)				
Health service navigation							
Cohort 1	3.2 (0.5)	3.2 (0.6)	3.3 (0.5)	.147	.017	.070	.023
Cohort 2 (post TTT-P)	3.1 (0.5)	3.3 (0.5)	3.2 (0.6)				
Social integration and support							
Cohort 1	2.6 (0.7)	2.7 (0.7)	2.8 (0.6)	**.020**	.033	.075	.022
Cohort 2 (post TTT-P)	2.7 (0.6)	2.8 (0.7)	2.7 (0.7)				
Emotional distress							
Cohort 1	2.7 (0.8)	2.3 (0.8)	2.3 (0.8)	**<.001**	.115	.474	.006
Cohort 2 (post TTT-P)	2.5 (0.7)	2.3 (0.9)	2.3 (0.7)				
General self-efficacy (GSE)							
Cohort 1	22.9 (6.0)	25.1 (6.9)	25.7 (6.3)	**<.001**	.135	.900	.001
Cohort 2 (post TTT-P)	23.6 (6.2)	26.0 (5.9)	26.2 (5.9)				
Health-related quality of life (WHOQOL-Bref)							
Physical health							
Cohort 1	50.3 (19.0)	62.7 (21.5)	60.9 (21.5)	**<.001**	.294	.371	.009
Cohort 2 (post TTT-P)	55.7 (19.0)	66.1 (20.9)	62.8 (19.8)				
Psychological health							
Cohort 1	41.8 (20.1)	56.0 (19.9)	51.7 (23.1)	**<.001**	.322	.678	.003
Cohort 2 (post TTT-P)	48.4 (21.1)	62.5 (22.1)	56.3 (21.0)				
Social relations							
Cohort 1	55.1 (20.4)	62.3 (19.5)	55.5 (21.3)	**.001**	.063	.843	.002
Cohort 2 (post TTT-P)	56.1 (23.4)	62.1 (21.3)	57.5 (23.1)				
Environment							
Cohort 1	64.2 (14.3)	68.6 (14.9)	69.3 (15.1)	**<.001**	.076	.368	.009
Cohort 2 (post TTT-P)	67.8 (14.7)	70.7 (13.9)	70.1 (13.7)				
Global quality of life							
Cohort 1	2.9 (0.9)	3.4 (1.0)	3.3 (1.0)	**<.001**	.184	.280	.011
Cohort 2 (post TTT-P)	3.0 (0.9)	3.5 (0.9)	3.2 (1.0)				
Satisfaction with quality of life							
Cohort 1	2.4 (1.1)	3.20 (1.0)	3.0 (1.1)	**<.001**	.296	.649	.004
Cohort 2 (post TTT-P)	2.4 (1.0)	3.26 (1.1)	2.9 (1.1)				

Abbreviations: SD: standard deviation; TTT-P: train-the-trainer program for healthcare professionals. Significant *p* values are marked in bold.

**Table 5:** Intermediate-term effects on secondary outcomes in cancer patients (*n* = 109).

Cancer patients	Beginning of the rehabilitation program	End of the rehabilitation program	6-month follow-up	Time		Time∗group	
	Mean (SD)	Mean (SD)	Mean (SD)	*p* value	Partial eta^2^	*p* value	Partial eta^2^
Self-management competence (heiQ®)							
Positive and active engagement in life							
Cohort 1	3.1 (0.5)	3.2 (0.6)	3.2 (0.5)	.**011**	.044	.281	.012
Cohort 2 (post TTT-P)	3.0 (0.7)	3.2 (0.5)	3.0 (.7)				
Health-directed activities							
Cohort 1	3.1 (0.8)	3.2 (0.7)	3.2 (0.7)	.136	.019	.866	.001
Cohort 2 (post TTT-P)	3.0 (0.8)	3.1 (0.6)	3.1 (0.9)				
Skill and technique acquisition							
Cohort 1	3.1 (0.5)	3.2 (0.5)	3.2 (0.5)	**.002**	.057	.127	.020
Cohort 2 (post TTT-P)	3.0 (0.6)	3.3 (0.5)	3.2 (.5)				
Constructive attitudes and approaches							
Cohort 1	3.4 (0.5)	3.3 (0.5)	3.3 (0.5)	.080	.024	.896	.001
Cohort 2 (post TTT-P)	3.5 (0.6)	3.4 (0.5)	3.4 (.6)				
Self-monitoring and insight							
Cohort 1	3.1 (0.5)	3.2 (0.4)	3.3 (0.4)	**.005**	.051	.660	.004
Cohort 2 (post TTT-P)	3.1 (0.4)	3.1 (0.4)	3.2 (0.5)				
Health service navigation							
Cohort 1	3.4 (0.6)	3.4 (0.5)	3.5 (0.4)	.384	.009	.206	.015
Cohort 2 (post TTT-P)	3.4 (0.4)	3.5 (0.4)	3.5 (0.5)				
Social integration and support							
Cohort 1	3.2 (0.7)	3.2 (0.6)	3.1 (0.6)	.401	.009	1.00	.370
Cohort 2 (post TTT-P)	3.4 (.6)	3.3 (0.6)	3.3 (0.5)				
Emotional distress							
Cohort 1	2.3 (0.7)	2.2 (0.7)	2.3 (0.8)	.238	.014	.401	.009
Cohort 2 (post TTT-P)	2.2 (0.8)	2.2 (0.7)	2.3 (0.8)				
General self-efficacy (GSE)							
Cohort 1	30.1 (4.1)	29.8 (5.3)	29.6 (5.0)	.271	.013	.441	.008
Cohort 2 (post TTT-P)	29.7 (4.8)	28.5 (5.7)	29.3 (5.0)				
Health-related quality of life (WHOQOL-Bref)							
Physical health							
Cohort 1	65.6 (17.6)	69.2 (17.5)	69.6 (18.6)	**<.001**	.084	.531	.007
Cohort 2 (post TTT-P)	62.9 (17.1)	68.3 (14.2)	70.2 (18.5)				
Psychological health							
Cohort 1	69.8 (16.2)	72.7 (16.3)	70.2 (16.0)	**.006**	.051	.911	.001
Cohort 2 (post TTT-P)	68.0 (18.1)	71.8 (16.2)	68.7 (20.8)				
Social relations							
Cohort 1	72.6 (17.3)	74.6 (17.9)	68.0 (19.6)	**<.001**	.079	.316	.012
Cohort 2 (post TTT-P)	70.1 (20.4)	75.0 (19.5)	69.9 (20.0)				
Environment							
Cohort 1	73.2 (12.0)	74.0 (12.1)	73.6 (13.8)	.196	.017	.437	.009
Cohort 2 (post TTT-P)	73.1 (12.7)	74.7 (12.7)	75.8 (13.0)				
Global quality of life							
Cohort 1	3.4 (.7)	3.6 (0.7)	3.6 (0.7)	**<.001**	.088	.693	.003
Cohort 2 (post TTT-P)	3.3 (.7)	3.6 (0.6)	3.5 (1.0)				
Satisfaction with quality of life							
Cohort 1	3.1 (1.0)	3.5 (0.7)	3.3 (0.9)	**<.001**	.109	.409	.008
Cohort 2 (post TTT-P)	3.0 (0.9)	3.4 (0.7)	3.3 (0.9)				

Abbreviations: SD: standard deviation; TTT-P: train-the-trainer program for healthcare professionals. Significant *p* values are marked in bold.

## Data Availability

The authors have full control over the primary data. The data analyzed in this study are housed at the Department of Medical Psychology, University Medical Center Hamburg-Eppendorf, Hamburg, Germany. According to the ethical committee approval, this data set is subject to ethical restrictions and local data protection regulations. Participants did not consent to have data made available for third parties. All relevant data for the conclusions are presented in the manuscript.
